# Withdrawal

**DOI:** 10.1002/fsn3.3089

**Published:** 2022-10-19

Withdrawal: “Spectral pattern study of citrus black rot caused by *Alternaria alternata* and selecting optimal wavelengths for decay detection,” by Narges Ghanei Ghooshkhaneh, Mahmood Reza Golzarian, and Mojtaba Mamarabadi, *Food Science & Nutrition*, 2022, 10, 1694–1706. https://doi.org/10.1002/fsn3.2739

The above article, published online in Volume 10, Issue 6 in Wiley Online Library (https://onlinelibrary.wiley.com/doi/full/10.1002/fsn3.2739), has been withdrawn by agreement between the Journal's Editor‐in‐Chief, Prof. Y. Martin Lo, and Wiley Periodicals, LLC. The withdrawal has been agreed because the article was published in error after having been rejected. The authors bear no fault, and we apologize for the error.

